# Preparation of Pt-Catalyst by Poly(*p*-phenylenediamine) Nanocomposites Assisted by Microwave Radiation for Proton Exchange Membrane Fuel Cell

**DOI:** 10.3390/polym10121388

**Published:** 2018-12-14

**Authors:** Yen-Zen Wang, Tsung-Han Ko, Wen-Yao Huang, Tar-Hwa Hsieh, Ko-Shan Ho, Yi-Yin Chen, Siang-Jhih Hsieh

**Affiliations:** 1 Department of Chemical and Materials Engineering, National Yun-Lin University of Science and Technology,123, Univ. Rd., Sec. 3, Douliou, Yun-Lin 64002, Taiwan; wangzen@yuntech.edu.tw; 2 Department of Photonics, National Sun Yat-sen University, 70 Lienhai Rd., Kaohsiung 80424, Taiwan; thk@yuntech.edu.tw; 3 Department of Chemical Engineering, National Taiwan University, 1, Sec. 4, Roosevelt Rd., Taipei 10617, Taiwan; wyhuang@faculty.nsysu.edu.tw (W.-Y.H.); thh@nkust.edu.tw (T.-H.H.); 4 Department of Chemical and Materials Engineering, National Kaohsiung University of Science & Technology, 415, Chien-Kuo Road, Kaohsiung 80782, Taiwan; rain19950409@gmail.com (Y.-Y.C.); abcd20256@gmail.com (S.-J.H.)

**Keywords:** poly(*para*-phenylenediamine), 5,10-dihydrophenazine, microwave assisted reduction, Pt, PEMFC

## Abstract

The Pt elements are prepared via the redox reaction with microwave (MW) irradiation in the presence of poly(p-phenylenediamine) (PpPD) which is polymerized on XC72 carbon matrix (PpPD/XC72), behaving as reducing agent. The free primary amines of PpPD are actually converted (oxidized) to secondary ones (5,10-dihydrophenazine) after MW irradiation. Transmission electronic microscopy (TEM) micrographs reveal the prepared Pt nanoparticles are well-dispersed on the carbon matrix like commercial Pt-implanted carbon nanocomposite (Pt/C). From the residue weights of thermogravimetric analysis (TGA) thermograms of Pt-loaded PpPD/XC72 (PpPD/XC72-Pt-MW), more Pt (18.49 wt %) nanoparticles are implanted on PpPD/XC72 composite. The Pt-implanted wt % on PpPD/XC72 matrix is just slightly lower than that of commercial Pt/C (22.30 wt %). The Pt-catalyst supports of PpPD/XC72-Pt-MW illustrate typical cyclic voltammograms (C-V) of Pt-catalyst, including significant Pt–H oxidation and Pt–O reduction peaks. The electrochemical active surface area of PpPD/XC72-Pt-MW is found to be as high as 60.1 m^2^ g^−1^. Max. number of electron transfer during oxygen reduction reaction (ORR) approaches 3.83 for PpPD/XC72-Pt-MW, higher than that of commercial Pt/C (3.62). Single cell based on PpPD/XC72-Pt-MW demonstrates much higher specific max. power density to be 34.6 mW cm^−2^ Pt, higher than that single cell prepared with commercial Pt/C electrode (30.6 mW cm^−2^ Pt).

## 1. Introduction

In order to reduce the greenhouse gas emissions and smog pollution [1,2,3] generated from fossil fuels, hydrogen-based fuel cell technologies have been widely studied in various areas [4].

Recent researches focus on hydrogen (anode) and oxygen (cathode) based proton exchange membrane fuel cells (PEMFCs), considering the environmentally friendly by-products of water and higher power density, lower noise and operating temperatures. Whereas, the major obstacle to commercialize PEMFCs is how to cost-down the fuel cell by improving Pt-catalytic efficiency and durability under the severe working conditions [5]. Other factors that related to the performance of the PEMFCs are dependent on the properties of the fuel gas (such as concentration of O_2_ in the cathode) flowing into the membrane electrode assembles (MEAs) [6], where lots of interfacial boundaries are present within the fuel gas, electrolyte and Pt-catalyst and the so-called triple-phase boundary plays decisive roles on the come-out power density.

Conducting carbon matrix materials are usually chosen as the catalyst-supporting materials for Pt-catalyst due to their excellent conductivity, high surface area and low cost, which are all necessary to reduce the cost of PEMFC. Some publications are focusing on creating nanoscale pores on the surfaces of conducting catalyst supports to trap and distribute the obtained Pt [7]. In most of the cases, Pt-catalyst supports for MEA are prepared by direct implanting nanoplatinum (Pt) particles on the surface of the conducting carbon black (Vulcan XC72). However, carbon support used in the cathode can be subjected to severe corrosion caused by the water by-product after ORR, which can very likely lead to the evolving poisoning carbon monoxide and carbon dioxide at high working temperatures [8,9,10], inducing fast decaying of the Pt catalysts and significantly curtailing the working hours of PEMFC. To construct Pt-catalyst carbon supporting materials with low corrosion at the working temperature has become an important topic in preparing PEMFC.

A core-shell N-containing polyaniline/XC72 composite was prepared by in-situ polymerization [11], to increase its resistivity to the CO-poisoning and improve the Pt-catalytic efficiency. And Pt implanted on N-containing carbon matrix is found to be more disperse, more electrochemically active and less particle ripening is caused compared with neat carbon matrix [12,13]. Lots of approaches were used to carbonize the nanostructured nitrogen-containing catalyst support and created new areas in the field of nitrogen-containing conducting carbon nanomaterials. Some fabricated an MEA based on polyaniline cathode which gained high power density with few Pt implanted [14]. N-containing nanosheet/nanotube with carbonized polyaniline on the carbon surfaces was synthesized as a carbonaceous support for Pt-catalyst [15], which revealed enhanced ORR in either acidic or alkaline media. Furthermore, amino-containing XC72 support is able to capture the Pt(IV) and the reduced Pt particles can be well-distributed on the supports after MW irradiation. N-containing carbon supports have been found to be able to enhance on ORR activity [16,17], which is related to the different morphologies and properties of the N-containing precursors it obtained from [18,19].

Besides, MW irradiation can be performed in a facile way in the absence of solvent, saving the troubles of drying sample and removing solvents. Especially, MW irradiation can be operated at any temperature with short time of exposure.

Since XC72 is known to own carbonyl groups on the surfaces, lots of types of oligoanilines which can act as both reducing agents and MW absorbing materials are H-bonding with XC72 before the addition of alcohol solution of hydrogen hexachloroplatinate (IV) hexahydrate (H_2_PtCl_6_·6H_2_O), followed by MW irradiation [20,21,22,23,24,25].

In our previous studies, N-phenyl-p-phenylenediamine (the simplest oligoaniline) was used as the reducing agent for Pt-catalyst prepared by MW irradiation [25]. It turned out to be successful and facile. However, much N-phenyl-*p*-phenylenediamine was lost during preparation due to its high solubility in alcohol solvents which are used during MEA preparation. And only few *N*-phenyl-*p*-phenylenediamine was placed on the surface of XC72, resulting in the high loss of Pt-particles in the solvent after MW irradiation.

In this study, we try to prepare a polymeric amino-containing XC72 as the Pt-catalyst by direct polymerizing cheap *p*-phenylene diamine (pPD) on the XC72 surface. The obtained PpPD will be firmly adhered on the XC72 (PpPD/XC72) without losing into the solvent during MEA or single cell fabrication due to its poor solubility. PpPD also owns free amino groups like *N*-phenyl-*p*-phenylenediamine, which can behave as both reducing agents and MW absorbers. Several properties like Pt-loading % of the catalyst support, ORR activity, electrochemical performance of the MEA and the power density of the single cell will be measured for the PpPD/XC72 composite based PEMFC.

## 2. Material and Methods

### 2.1. Materials

The ammonium persulfate (Merck KGaA, Darmstadt, Germany), hydrogen hexachloroplatinate (IV) hexahydrate (H_2_PtCl_6_·6H_2_O, Merck KGaA, Darmstadt, Germany), p-phenylenediamine (TOKYO KASEI KOGYO CO., Tokyo, Japan), ethylene glycol (EG, J.T. Baker, PA, USA), Vulcan XC72 (Cabot, Massachusetts, USA) were applied without further purification.

### 2.2. Preparation of PpPD/XC72-Pt-MW

9 mg of pPD was polymerized on 16 mg of XC72 by aqueous ammonium persulfate (APS). The obtained PpPD/XC72 composites were mixed with the same amount of hexachloroplatinate (H_2_PtCl_6)_ acid (0.025 mmole) in 10 mL of ethylene glycol (EG). The mixture was under sonication until uniform and was neutralized with 9 ml, 1 M NaOH(aq) keep the pH value at 11 and subjected to MW irradiation for 100 s (the energy input value is 700 W × 100 s = 70,000 J. The obtained PpPD/XC72-Pt-MW composites were separated by direct filtration and washed with de-ion water followed by drying in an oven at 60 ℃ overnight. Comparison MW irradiation redox reaction experiments was carried out in the absence of either PpPD or XC72.

### 2.3. Basic Characterization

#### 2.3.1. Microwave Oven

All samples were exposed to MW irradiation from a microwave oven (TMO-17MB model) with 2.45 GHz, made by Tatung co. (Taipei, Taiwan) with max. power of 700 W.

#### 2.3.2. FTIR Spectroscopy

The functional groups of neat PpPD and PpPD/XC72 composite before and after MW irradiation were obtained from FTIR spectra which were recorded on an IFS3000 v/s FTIR spectrometer (Bruker, Ettlingen, Germany) at room temperature with a resolution of 4 cm^−1^ and 16 scanning steps.

#### 2.3.3. X-ray Photoelectron Spectroscopy (XPS)

The different binding energy spectra of N1s of PpPD-Pt-MW and PpPD/XC72-Pt-MW were used to characterize the percentage of primary, secondary amines and anilinium salt after MW irradiation by an XPS instrument of Fison (VG)-Escalab 210 using Al Ka X-ray source at 1486.6 eV. The pressure in the chamber was maintained under 10^−6^ Pa or lower during the measurement. A tablet sample was prepared by a stapler. The binding energies of the N1s around 400 eV were recorded.

#### 2.3.4. TEM

Samples taken photos by field emission transmission electron microscope, HR-AEM (HITACHI FE-2000, Hitachi, Tokyo, Japan) were first dispersed in acetone and put on carbonic-coated copper grids dropwise before subjecting to the emission.

#### 2.3.5. TGA

The residue weights of thermal degradation of neat PpPD and PpPD/XC72 composite before and after MW irradiation was recorded by TGA (TA SDT-2960, New Castle, DE, USA) thermograms. The amount of Pt loaded were characterized by the residual weights at 800 °C at 10 °C min^−1^ with purging air.

#### 2.3.6. WXRD (Wide Angle X-ray Diffraction: Powder X-ray Diffraction)

A copper target (Cu-Kα) Rigaku X-ray source with a wavelength of 1.5402 Å was used for X-ray diffraction. The scanning angle (2θ) started from 5° to 40° with a voltage of 40 kV and a current of 30 mA, operated at 1° min^−1^.

### 2.4. Electrochemical Characterization

Cyclic voltammetry method was used to determine the active electrochemical surface area of the catalyst supports in the electrode. The performance of the electrocatalyst support was tested with a three-electrode system. The round working electrode with an area of 1.5 cm^2^ was prepared as follows. Ag/AgCl and carbon graphite were used as the reference and relative electrode, respectively. The electrochemical test was carried out in a potentiostat/galvanostat (Autolab-PGSTAT 30 Eco Chemie, Utrecht, Netherlands) in 0.5 M H_2_SO_4_ solution and C-V curves were obtained with scanning potential from −0.2 to 1.0 V at a sweeping rate of 50 mV s^−1^. The catalyst ink was prepared by mixing 3mg support powder in isopropanol and stirred until it became uniform. Subsequently, 5% Nafion solution was added into the mixture as binder and the mixture was ultra-sonicated for 1 h, the obtained ink was uniformly spray-coated on the carbon paper for C-V test.

The polarization curves of the PpPD/XC72-Pt-MW were measured using a rotating-disk electrode (RDE) operated at 900, 1200, 1600, 2500 and 3600 rpm for in O_2_-saturated 0.5 M H_2_SO_4_, respectively. The ORR currents of XC72-Pt-MW and PpPD/XC72-Pt-MW were recorded at the measured voltage range (−1.2–0.5 V) with 1600 rpm.

### 2.5. MEA Preparation

A Nafion® 212 sheet purchased from Ion Power Inc., New Castle, DE, USA was used as the proton exchange membranes. In order to remove the surface organic impurities and to convert the membranes into protonated (H^+^) form, the Nafion-212 (4 × 4 cm^2^) membrane was treated at 70 ℃ in 5 wt % H_2_O_2_ aqueous solution for 1 h, followed by submerging in 1 M H_2_SO_4_ solution for 1 h and subsequently the treated membranes were dipped in distilled water for 15 min and stored in de-ionized water. The catalyst inks were prepared by mixing 20 mg of PpPD/XC72-Pt-MW (or XC72-Pt-MW, PpPD-Pt-MW) powders in isopropanol and mechanically stirred until it became uniform before 5% Nafion solution was added. Eventually, the catalyst mixture was ultra-sonicated for 1h followed by coating on both side of the treated Nafion sheet dropwise as anode and cathode electrodes (2 × 2 cm^2^), respectively and hot-pressed at 140 °C with a pressure force of 70 kg cm^−2^ for 5min to obtain the MEA.

### 2.6. Single-Cell Performance Testing

The MEA was installed in a fuel cell test station for testing using the single-cell test equipment (model FCED-P50; Asia Pacific Fuel Cell Technologies, Ltd., Miaoli, Taiwan). The active cell area was 2 × 2 cm^2^. The temperatures of anode, cell and cathode and humidifying gas were all maintained at around 70 °C. The flow rates of anode input H_2_ and the cathode input O_2_ fuels were set at 200 and 100 mL·min^−1^, respectively, based on stoichiometry. To test the electrochemical performance of XC72-Pt-MW, PpPD-Pt-MW and PpPD/XC72-Pt-MW catalyst in the individual MEAs, both C-V and output powers were measured.

## 3. Results and Discussion

### 3.1. Microwave Absorption of PpPD

The oxidation of one of the amino- group of pPD monomers can only be performed on the strongly acidic condition by strong oxidant like persulfates and the monomers can be polymerized (oxidized) into PpPD [26,27,28] and the remaining amine group which can perform redox reaction with Pt ions under MW irradiation. Usually, a strong acidic condition are created after Pt-reduction due to lots of protons are released (by-products) from PpPD. The proton products can be neutralized by the input aqueous sodium oxide (pH = 11) in the PtCl_6_^−2^ aqueous solution to avoid the reverse redox reaction and to improve the yield of Pt elements. The redox reaction can also be improved by the rising temperature by the generated heat evolved from the MW-absorption of the amino groups of PpPD which behaves as the reducing agent at the same time.

One of the amino groups of PpPD is considered as an effective microwave absorber compared to the poor MW absorption capability of alcohol type of chemicals like EG (actually no significant Pt-NPs were found in the absence of PpPD under MW irradiation). The reducing capability of PpPD can be activated by the microwave absorption, which can activate the redox reaction between PpPD and Pt ions under MW irradiation. The following Pt-catalysts were prepared in a regular microwave oven with tunable power (700 W max.) and a frequency of 2.45 GHz.

**Scheme 1:**
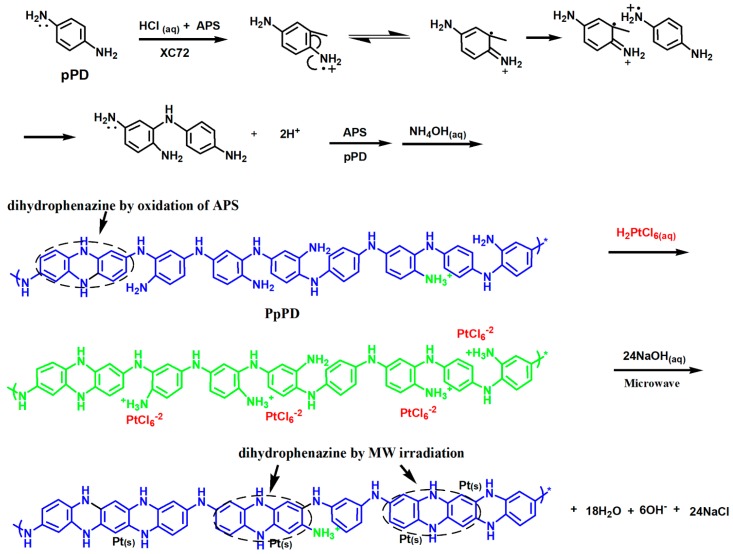
Polymerization of PpPD and MW assisted redox reaction between PtCl_6_^−2^ and PpPD.

**Scheme 2:**
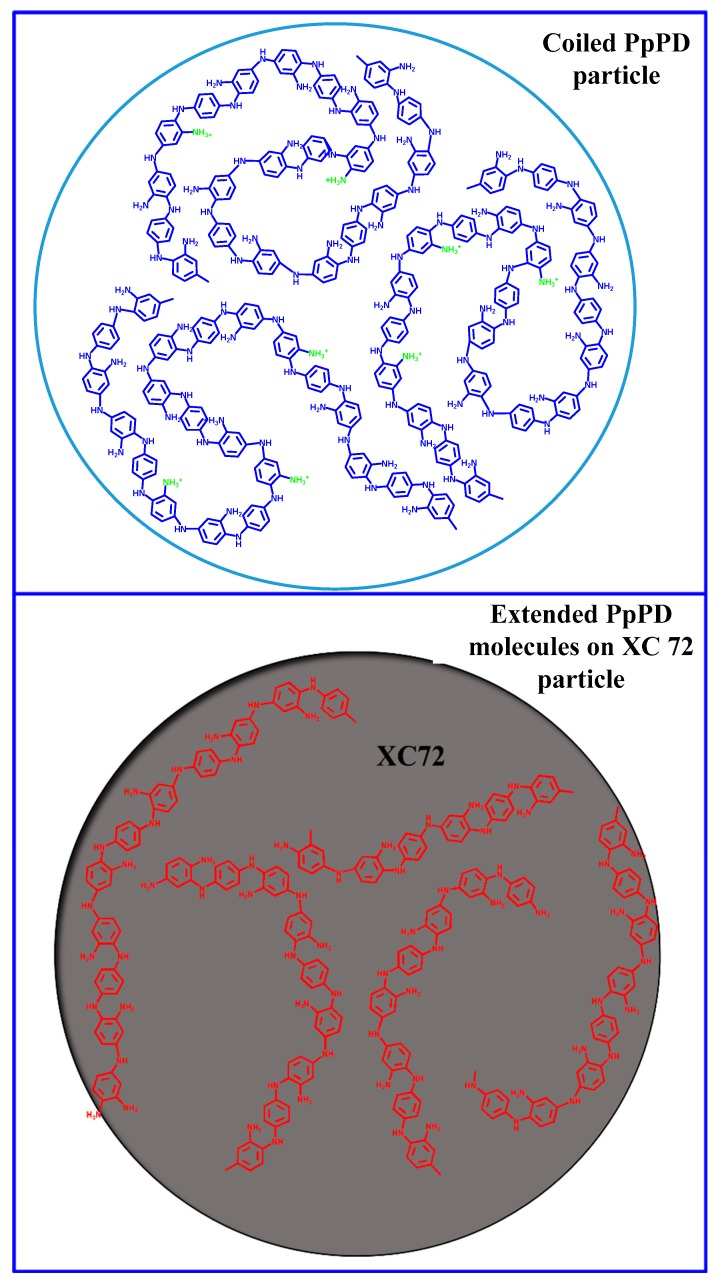
Schematic diagram of coil (neat) PpPD particle and extend PpPD on XC72 particle.

Another purpose of applying PpPD as the reductant is its remaining free amine groups after polymerization can chelate with Pt ions, fastening the process of coordination-oxidation nucleation [29] once MW is absorbed. Before MW irradiation, the surface of PpPD/XC72 particles immediately became dark when mixed in the stirring H_2_PtCl_6_ aqueous solution, indicating the chelation of Pt ions to the amine groups of PpPD. The PpPD in the complexes execute the reducing power to obtain Pt-NPs in 100 s of MW irradiation. And most of the primary amines of PpPD were converted into secondary ones connected with the neighboring benzene ring, which transformed PpPD into a ladder like polymer after MW irradiation depicted in Scheme 1. The Pt quickly accumulated into spherical NPs and combined tightly on the PpPD/XC72 matrix as described in Scheme 2.

### 3.2. FTIR Spectroscopy

**Figure 1:**
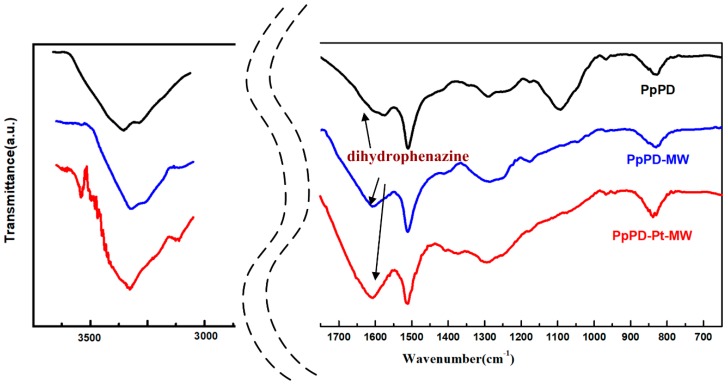
FTIR spectra of neat PpPD and Pt/XC72-PpPD-MW.

**Scheme 3:**
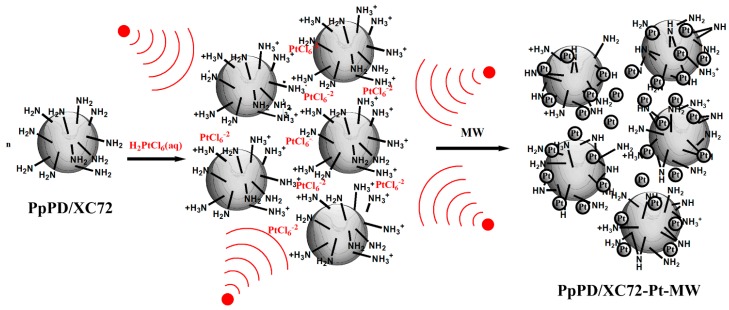
Schematic diagram of PpPD/XC72-Pt-MW preparation.

The FTIR spectra of neat PpPD, MW-irradiated PpPD and Pt-complexed PpPD are demonstrated in Figure 1 which clearly demonstrate the doublet peaks of primary amine of neat PpPD around 3300 cm^−1^. Double peaks still partly remain if PpPD is irradiated with MW in the absence of Pt ions, revealing that absorbed MW by the amine groups was not used to induce electrochemical reaction but dissipated into heat. The doublet peaks of primary amine become like singlet peak after MW irradiation in the presence of Pt ions. In other words, Pt ions join in the redox reaction and convert some of the primary amine into secondary one after MW assisted oxidation (Scheme 3). It means the Pt ions were successfully reduced into element state by primary amines of PpPD with the assistance of MW irradiation, depicted in Scheme 1. The proton by-products of the redox reaction were immediately neutralized by the present NaOH (pH = 11) to inhibit the reverse reaction according to Scheme 1 and the yield of Pt elements can be enhanced. The peak of 5,10-dihydrophenazine (dihydrophenazine) assigned at 1612 cm^−1^ (stretching vibration of conjugated C=C of benzene ring) [30,31] were found for all samples demonstrated in Figure 1. They can be created by the intramolecular oxidation between the amino groups and the neighboring aromatic H– during either oxidative polymerization or MW irradiation. When the oxidation continues, the peak of dihydrophenazine grow slightly under MW irradiation (Figure 1). The relative ratio of peak of dihydrophenazine to the neighboring peak at 1500 cm^−1^ (N–H deformation mode) is the highest for PpPD-Pt-MW compared to either neat PpPD or PpPD-MW according to Figure 1, indicating the dihydrophenazine is also the by-product of the Pt formation reaction as depicted in Scheme 1 and Figure 1. The MW irradiation also cause other reactions that are not related to the redox reaction. For example, the peak of neat PpPD centered 1164 cm^−1^ which is assigned to be the out of plane bending of –C–H group of benzene ring disappears after MW irradiation. However, peak at 820 cm^−1^ assigned to be the 1,2,4-substituted benzene rings of dihydrophenazine remains intact for PpPD-MW and PpPD-Pt-MW referring to Figure 1, revealing the polyaromatic structure of PpPD is not destroyed by either MW irradiation or redox reaction.

### 3.3. X-ray Photoelectron Spectroscopy (XPS)

**Figure 2:**
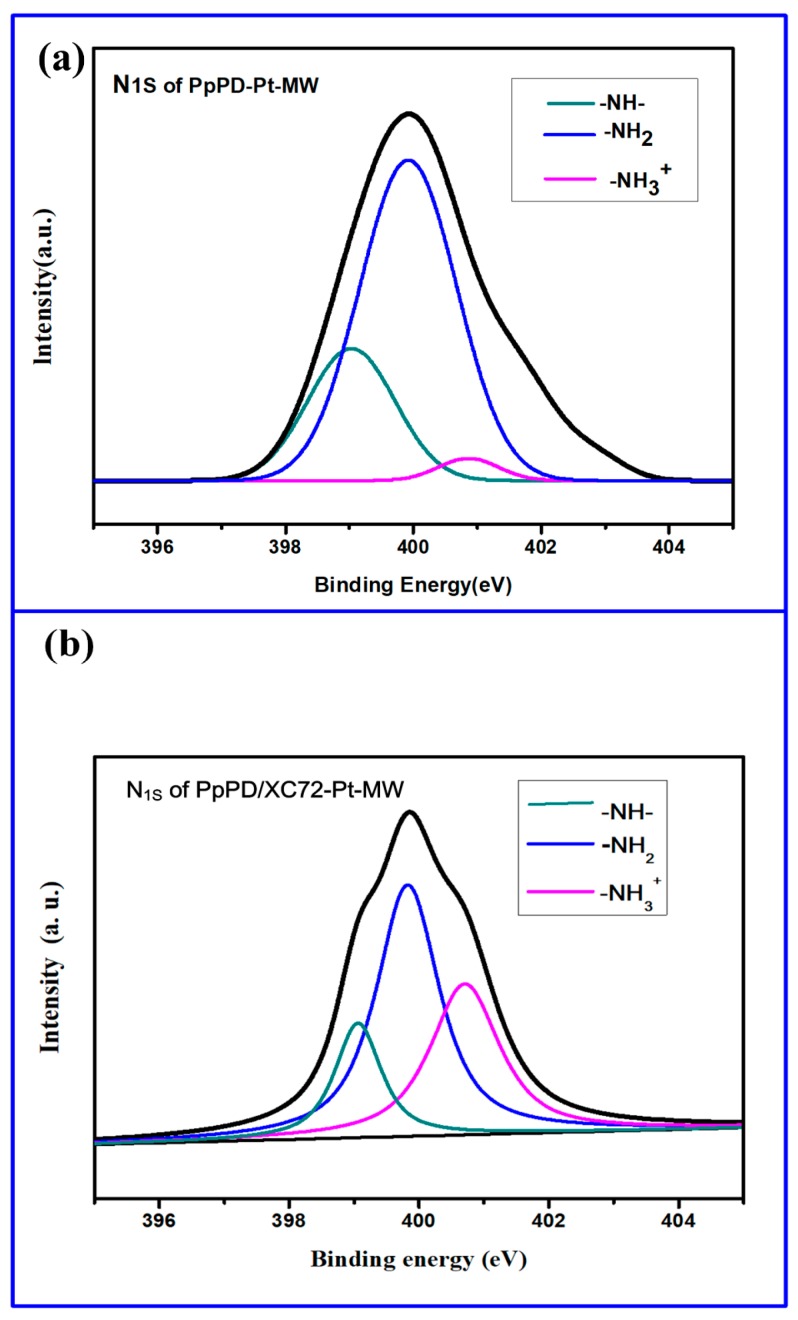
N1s of XPS of PpPD-Pt-MW and PpPD/XC72-Pt-MW.

To characterize furthermore on the PpPD after MW irradiation (PpPD-Pt-MW), N_1s_ of XPS was used to find out the various N-related functional groups created after polymerization. Three types of aminos centered at around 399, 400 and 401 ev were assigned as secondary, primary amine and ammonium, respectively, in Figure 2a. Figure 2a was de-convoluted into three individual peaks, representing as –NH–, –NH_2_– and –NH_3_^+^, respectively. Their quantities are obtained by the individual integrated areas which are listed in Table 1. We understand there are still lots of primary amines remained for PpPD-Pt-MW after MW irradiation, which could be used a reducing agent for Pt ions assisted with MW irradiation. After doping by H_2_PtCl_6_, the ammonium salts were created and we can see the peak at 401 eV increase significantly in the N1s of XPS of PpPD/XC72-Pt-MW demonstrated in Figure 2b. The redox reaction which results in the production of Pt elements mostly carried out on the primary amine of PpPD, leading to the formation of dihydrophenazines according to Figure 1 and previous discussions. We believe more secondary amine of dihydrophenazine were created when PpPD was prepared on the surface of XC72 (PpPD/XC72-Pt-MW), which enhanced the secondary/primary amino ratios listed in Table 1 from 0.37 to 0.96. The additional secondary amine ratio (0.96 − 0.37 = 0.59) in PpPD/XC72-Pt-MW is believed to be contributed from the dihydrophenazines of the PpPD molecules that were polymerized on the XC72 surface, which are able to expose more primary amines to Pt ions. In other words, more dihydrophenazine created, more Pt elements can be obtained for PpPD/XC72-Pt-MW. Therefore, the production of Pt elements can be enhanced, which will be discussed in the thermogravimetric analysis.

### 3.4. TEM

Generally, the catalytic efficiency of the implanted Pt particles which allows redox reaction to proceed on their surface active sites is strongly dependent on its dispersibility on the conducting substrate. The Pt needs to be prepared in a way that Pt-particles can be well-separated from each other to provide as much active sites as possible for the redox reaction.

**Figure 3:**
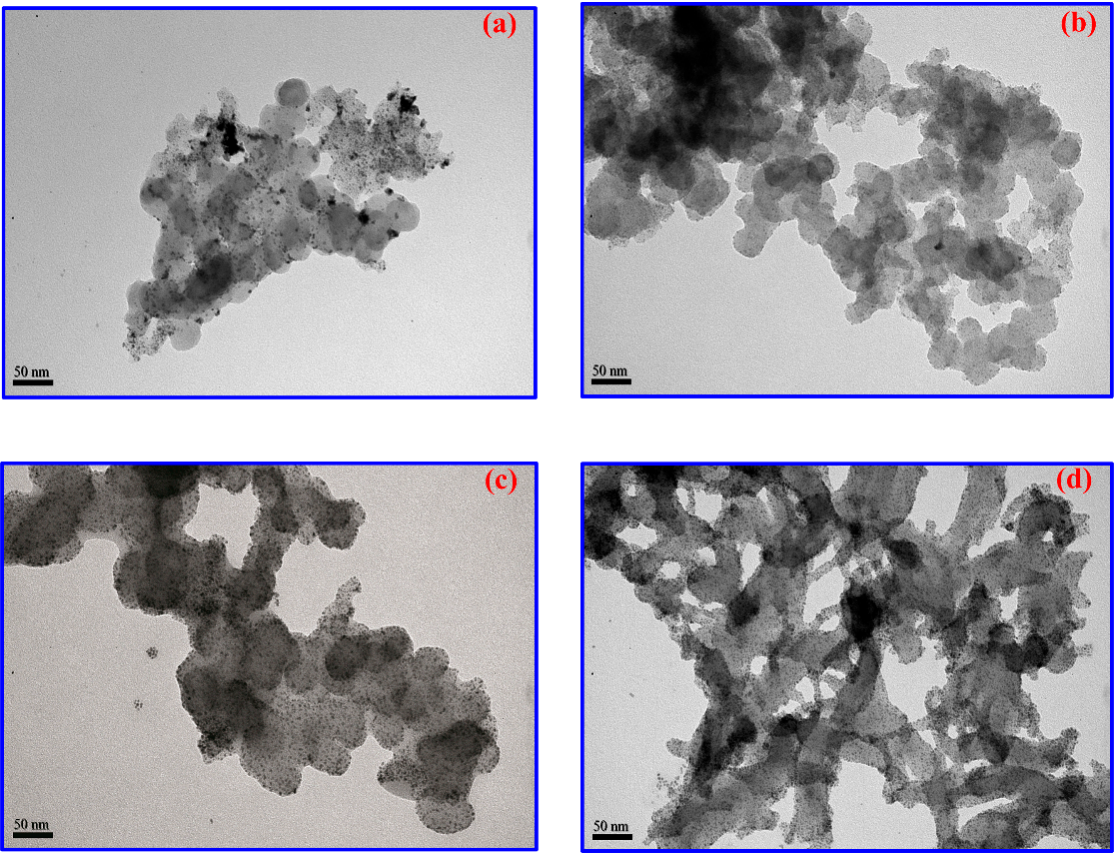
TEM micrographs of various substrates after MW irradiation (**a**) PpPD-Pt-MW (**b**) XC72-Pt-MW (**c**) PpPD/XC72-Pt-MW (**d**) commercial Pt/C.

The distribution and particle size of Pt-nanoparticles (Pt-NPs) obtained from MW irradiation on various substrates can be seen from the TEM micrographs demonstrated in Figure 3 which significantly demonstrates the locations and degree of accumulation of the implanted Pt-NPs. Clearly, the Pt-NPs are seriously aggregated into larger, micro-size particles if no substrate is present during MW assisted reduction (Figure 3a). When the neat PpPD molecules were prepared in the absence of any matrix, they easily coiled into large, micro-size particles (Scheme 2) with less surface area to accommodate the Pt ions. Besides, the functional amino groups which would capture (complex) Pt ions are easily imbedded inside of neat PpPD particles, which makes the redox reaction leading to the formation of Pt-NPs more difficult to perform. When there is no PpPD polymerized on the XC72 surface to capture Pt ions during MW irradiation, all the Pt-NPs can only be obtained with the help of the heat generated by MW, similar to hydrothermal method. The yield of Pt-NPs becomes very small although they own smaller sizes and are well dispersed on the surface of neat XC72, referring to Figure 3b. Besides, there is no attracting force to immobilize Pt ions and to create Pt-NPs onto the XC72 surfaces. The Pt-NPs are only accidentally loaded on during MW irradiation.

After PpPD molecules were polymerized and extendedly laid on the surface of XC72 (Scheme 2), the PtCl_6_^−2^ ions (H_2_PtCl_6_) can complex with the ammonium (–NH_3_^+^) ion which is the other product from the neutralization reaction between H_2_PtCl_6_ and primary amines of PpPD (Scheme 1). In other words, Pt ions in the form of PtCl_6_^−2^ can firmly graft on XC72 indirectly through the PpPD bridge (Scheme 3) which is PpPD/XC72 nanocomposite. Above all, the primary amines of PpPD are strong reducing agents for Pt-reduction under MW irradiation and the obtained Pt-NPs can well-disperse on the PpPD/XC72 surface as seen in Figure 3c. Comparing with the TEM micrograph of commercial Pt/C in Figure 3d, Pt-PpPD/XC72 demonstrates very similar way of Pt-NP dispersibility and particle size distribution.

The exact weight of the Pt-loading % will be obtained from the TGA thermograms in the following discussion.

### 3.5. TGA

**Figure 4:**
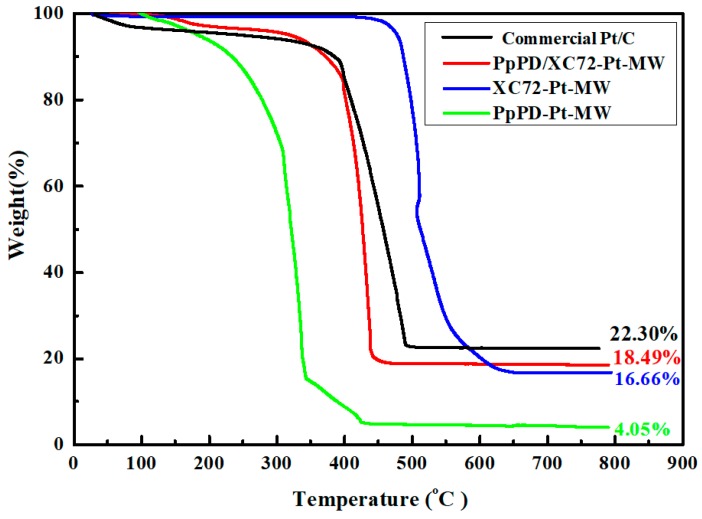
TGA thermograms of various Pt-loaded catalysts.

The efficiency of Pt-catalyst depends on both loading % and active surface area. All the organic and carbon components of the formed Pt-catalysts can be removed by high temperature burning and only Pt elements can be remained, which allows us to measure the Pt-loading % on the supports. Therefore, the residual weight obtained from the TGA thermogram can be interpreted as the Pt-loading % of various types of Pt-catalyst. It is found (Figure 4) the weight loss of all Pt-catalysts became stable after 650℃ and the flat (residue) weight % is defined as the Pt-loading % of the catalyst-supports.

The TGA thermogram in Figure 4 illustrates the Pt-loading % of PpPD-Pt-MW is 4.05 wt % lower than 10 wt %. Since only surface ammonium groups of PpPD particles are able to capture Pt ions to proceed redox reaction, the yield of Pt-NPs after MW irradiation is definitely very small. For XC72-Pt-MW, there are 16.66 wt % of Pt-NPs grafted and more Pt-NPs are prepared when PpPD is polymerized on its surface (18.49 wt %). Nearly 2 wt % (18.49 − 16.66 = 1.83 wt %) more are grafted onto XC72 matrix. Commercial Pt/C demonstrates the highest Pt-loading of 22.3 wt % as seen in Figure 4. However, higher Pt-loading % does not mean higher power when they are assembled into MEA and single cell. The active surface area of electrode-catalysts and the effect of ORR which will be measured and discussed on the following sections are two important factors which decide the eventual performance of a single cell.

### 3.6. XRD Pattern of the Electrocatalyst Electrode Materials

**Figure 5:**
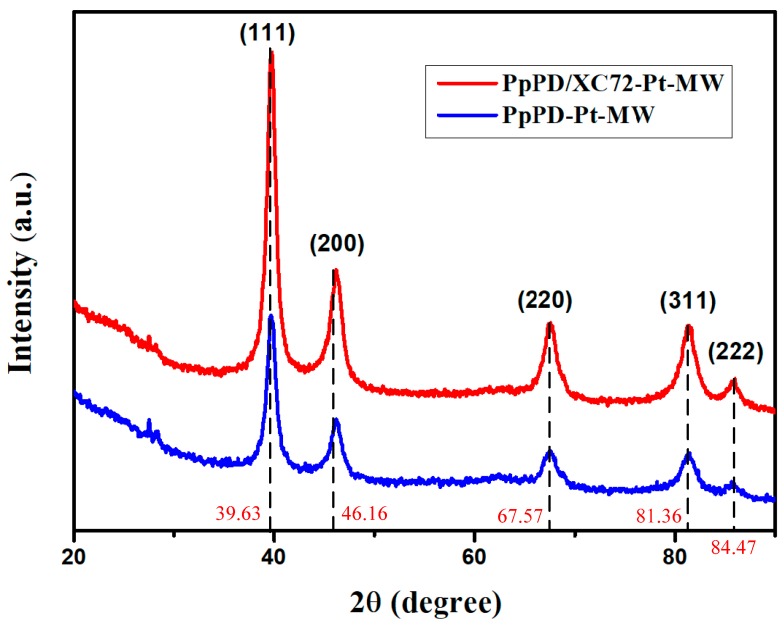
X-ray diffraction patterns of PpPD/XC72-Pt-MW and PpPD-Pt-MW.

The XRD patterns of Pt-crystalline loaded on both neat PpPD and PpPD/XC72 supports are demonstrated in Figure 5. Both demonstrate the feature diffraction peaks (FCC) of Pt crystalline at (111), (200), (220), (311) and (222), respectively, indicating the Pt can be prepared under MW irradiation in the presence of PpPD and the obtained Pt-crystals demonstrate the same type of crystalline structure like common Pt elements.

The mean crystallite sizes of Pt implanted on various substrates can be calculated from XRD patterns and are listed in 1st column of Table 2. It is found the particle sizes obtained are similar to those obtained from the TEM micrographs in Figure 3.

### 3.7. Electrochemical Analysis

#### 3.7.1. C-V

To evaluate the activity of Pt-catalyst, the C-V curves were constructed by sweeping potential from −0.2 to 1.0 V by normal hydrogen electrode with a scan rate of 50 mV s^−1^ at room temperature.

**Figure 6:**
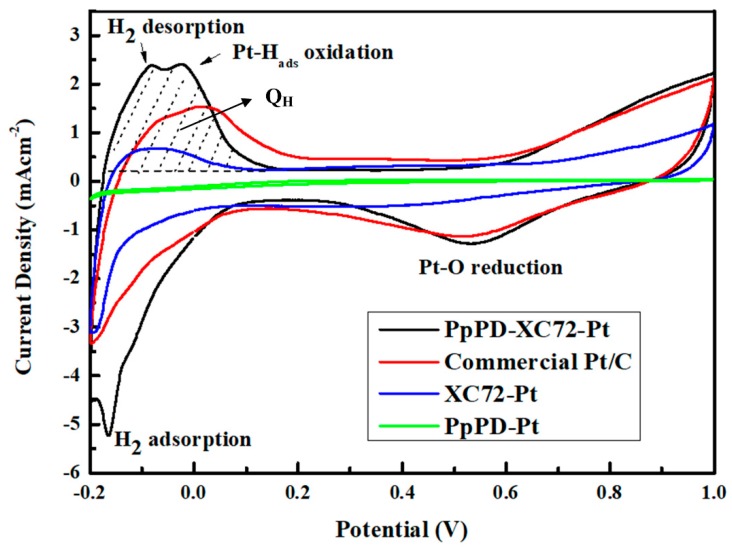
The current-voltammogram curves of various Pt-electrodes.

The electrochemical active surface area (ECSA), representing the Pt-catalyst activity is measured by the total hydrogen oxidation charge (Q_H_: the integration area of hydrogen desorption peak) illustrated in Figure 6 and divided by the weight of loading Pt-catalyst [32,33]. The obtained ECSA for various Pt-catalysts are listed in Table 2. Obviously, Pt implanted on neat PpPD does not provide any electrochemical activity since only 4.05% of Pt are loaded. The less surface area of PpPD particles of coiler molecules (Scheme 2) results in no significantly measurable ESCA seen in Figure 6 and Table 2. The ESCA of Pt is 27.5 m^2^ g^−1^ if XC72 is used to replace PpPD as supporting materials during MW irradiation. However, its C-V curve does not show significant Pt–H adsorption of oxidation (smaller Q_H_). After PpPD has been placed (polymerized) on the surface of XC72, the mobile Pt ions are able to graft on and complexed with the –NH_2_ groups of extended PpPD molecules before MW irradiation. Clearly, the presence of primary amines can effectively provide lots of surface capturing sites for Pt ions by the strong attraction between –NH_3_^+^ and PtCl_6_^−2^ ions. In the absence of PpPD (primary amines), less than half ESCA (Table 2) is obtained for XC72-Pt-MW since no significant interaction is present between PtCl_6_^−2^ and XC72 matrix.

Therefore, more Pt elements are reduced and implanted on the surfaces of PpPD/XC72 nanocomposite, which can significantly raise the ECSA to 60.1 m^2^ g^−1^ (Table 2) and greatly improve the hydrogen oxidation capability (higher Q_H_) of the Pt-catalyst in Figure 6. Although the commercial Pt/C owns higher wt % of grafted Pt-NPs (Figure 4), it just demonstrates an ECSA of 30.5 m^2^ g^−1^ in Table 2 due to less hydrogen desorption and oxidation (less Q_H_), which is half of that of PpPD/XC72-Pt-MW.

Besides, the Pt ions captured by surface PpPD which covers on XC72 carbon matrix can be directly reduced and implanted on the N-containing XC72 matrix. The Pt nanoparticles is distributed uniformly on the carbon matrix and is more electrochemically active compared to those Pt implanted on empty voids and causes less particle ripening than neat carbon matrix [12,13].

The mass activity (MA) of Pt-supports can be obtained from multiplying the ECSA with the half-wave potential (*V*_1/2_) and listed in the 4th column of Table 2. The specific reduced current that follow through the PpPD/XC72-Pt-MW per mg is 1226 mA which is almost three times equal to that of XC72-Pt-MW (456 mA per mg of Pt) and almost two times equal to that of commercial Pt/C (707 mA per mg of Pt). The MA value demonstrates more significant specific activity for Pt-catalyst if they are prepared on the surface of PpPD/XC72 under the MW irradiation.

The C-V curve of PpPD/XC72-Pt-MW also demonstrates significant ORR peak (Pt-O) at around 0.6 V [34] compared to that of XC72-Pt-MW or PpPD-Pt-MW. There is no significant ORR for XC72-Pt-MW around 0.6 V even though some of the hydrogen is desorbed and oxidized, revealing ORR step has already become the barrier step (rate determining step) for the redox reaction.

#### 3.7.2. Linear Sweep Voltammetry (LSV) and Koutecky-Levich (K-L) Plot

Electrochemical testing also indicated that the Pt/XC72-PpPD-MW catalyst can significantly enhance the ORR [35,36] due to the large amount of well-dispersed Pt nanoparticles which were obtained from the reduction of amino-captured Pt ions which are reduced and implanted on the XC72 surface after the irradiation of MW.

Another approach to demonstrate the ORR of the catalyst is to measure the polarization curves of Pt-catalyst coated GC electrode at different disk rotation rates of the electrode (RDE) performed with LSV method.

**Figure 7:**
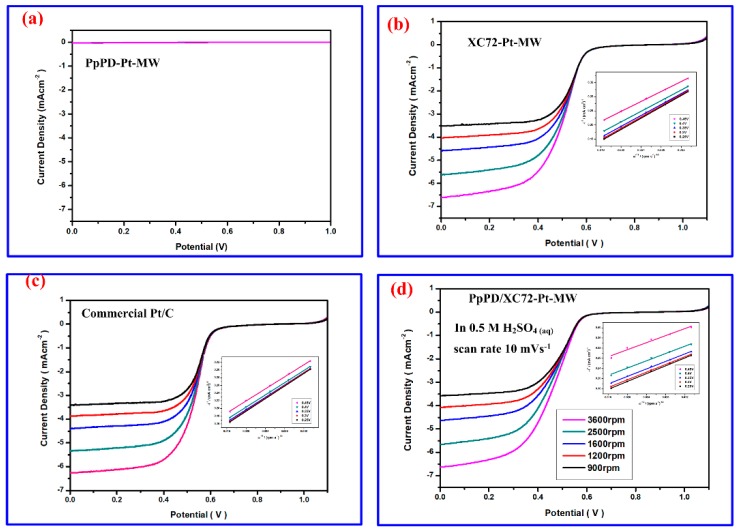
LSV curves and K-L plots of various Pt-electrodes at different rotating rates.

The polarization curves of various Pt-supports are illustrated in Figure 7. Almost no reduced current (electrochemical activity) can be found for PpPD-Pt-MW (Figure 7a), which has been explained in the previous discussion. Reduced currents are found when XC72 was used as the supporting substrate during Pt-reduction by MW irradiation and they increased with electrode-rotating speeds (Figure 7b). In the beginning (1.0–0.6 V), the current is controlled by the concentration (proportional to O_2_ partial pressure in the air) of O_2_ in the cathode, the so-called kinetic control. The kinetic current (*I*_kin_) is almost independent of rotating speed. When potential is decreased to less than 0.6, the diffusion-limited factor comes into effect and dominated below 0.4 V. At this stage, the diffusion current (*I*_dif_) significantly varies with the rotating speed, that is, the *I*_dif_ increased with rotating speed. We can choose the diffusion-controlled stages (0.15–0.4 V) and use the *I*_dif_ to construct the K-L plots at each voltage, which allow us to calculate the numbers of electrons transferred during ORR.

The LSV curves used to construct the K-L plot (inset figures) are demonstrated in Figure 7, which were then used to calculate the numbers of electron transfer during ORR at different potentials and listed in Table 3.

The max. number of electron transferred in ORR for XC72-Pt-MW and commercial Pt/C is 3.75 and 3.62, respectively and that of PpPD/XC72-Pt-MW is 3.83, listed in 1st column of Table 3. The max. number of electron transferred in ORR for XC72-Pt-MW is closer to the theoretical number (4) of the fully ORR in Equation (1), following a four-electron reduction model of O_2_, indicating less intermediate product (H_2_O_2_) is produced during ORR.

**O_2_ + 4H^+^ + 4e^−^ → 2H_2_O** (1)

The incomplete ORR reactions include reactions of Equations (2) and (3) which are series reactions during which O_2_ is reduced via two different steps. And the intermediate product, H_2_O_2_, is produced and consumed during these two incomplete ORRs.

**O_2_ + 2H^+^ + 2e^−^ → H_2_O_2_** (2)

**H_2_O_2_ + 2H^+^ + 2e^−^ → 2H_2_O** (3)

In each step, only two electrons are transferred and if some of the hydrogen peroxide are not further reduced in the next step, the averaged numbers of e-transferred will be less than 4. In other words, the degree of ORR can be represented by how close the max. number of e-transferred is to the ideal value of 4. In the present case, the max. number of e-transferred for PpPD/XC72-Pt-MW (3.83) is only slightly less than 4, demonstrating an almost complete ORR. For XC72-Pt-MW and commercial Pt/C, the max. number of e-transferred is 3.75 and 3.62, respectively due to more produced H_2_O_2_, resulting from the incomplete ORR of the Pt-catalyst in the cathode.

No significant H_2_O_2_ inducing *I*_dif_ fluctuation can be found in Figure 7 for all Pt-electrodes. More reduced current is obtained for PpPD/XC72-Pt-MW comparing with other Pt-electrodes in Figure 7, especially when the rotating speed is higher than 1600 rpm.

#### 3.7.3. Single Cell Performance Analysis

**Figure 8:**
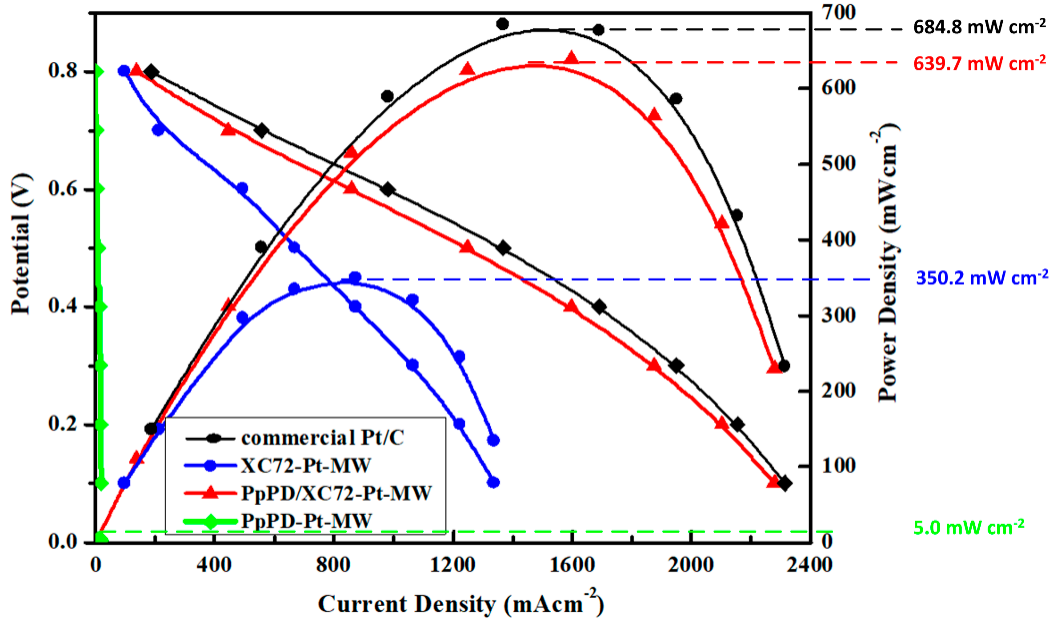
Electrocatalytic polarization and power density curve of PEMFC based on different catalyst supports. The fuel cell temperature was at 70 °C. The flow rates of H_2_ and O_2_ flows are set at 0.1 L min^−1^ and 0.2 L min^−1^, respectively.

MEAs with various Pt-catalyst electrodes are assembled into single cells and their electrochemical performances are evaluated by measuring their current density, voltage and power density in Figure 8. The presence of PpPD/XC72 nanocomposite during microwave reduction of Pt is the key factor deciding the final electrochemical performances of the resultant single cell.

When only neat PpPD was present in microwave-assisted reduction, the assembled single cell demonstrated almost zero max. power density (*P*_max_) and max. reduced current (*I*_max_). When PpPD was replaced by XC72 in the preparation of Pt-catalyst by MW irradiation, the *P*_max_ is increased to 350 mW cm^−2^ at an *I*_max_ of 1400 mA cm^−2^. After normalizing by the wt % of Pt-NPs obtained from TGA thermogram (Figure 4), the specific *P*_max_ is 21 mW cm^−2^_Pt_, listed in the 4th column of Table 3. Surprisingly, when MEA is prepared by the PpPD/XC72-Pt-MW which is obtained from polymerizing PpPD on the XC72 surface before MW irradiation, the *P*_max_ and specific *P*_max_ rise by 180% (639.7 mW cm^−2^) and by 165% (34.6 mW cm^−2^_Pt_) according to the 3rd and 4th columns of Table 3, facilitated mainly by the effective ORR. Even though commercial Pt/C has more Pt-loaded (22.3%) and demonstrates higher *P*_max_ (684.8 mW cm^−2^), its specific *P*_max_ (30.6 mW cm^−^^2^_Pt_) is less than that of PpPD/XC72-Pt-MW, resulting from poorer ORR. The presence of PpPD/XC72 nanocomposite in the microwave-assisted reduction of Pt can effectively mitigate the effect of concentration polarization, referring to Figure 8 and Table 3. And the cell went through only slight voltage drop with current density, contributed from the insignificant concentration polarization of the N-containing PpPD. Owning high specific *P*_max_ and *I*_max_ of the PpPD/XC72-Pt-MW based single cell indicate that we have already provided a facile approach to obtain efficient, large amount of Pt on the catalyst support by placing (polymerizing) the amino-containing polymers on the XC72 surface before microwave irradiation and surface amino-groups on the nanocomposite serve multi-purposes as reducing, chelating and MW-absorbing agents.

Although mass activity shown in RDE measurements for the PpPD/XC72-Pt-MW is better, the poorer interfaces during the fabrication did play an important part. Covering XC72 particles with PpPD did create addition interfacial problems which do not exist for XC72-Pt or commercial Pt/C.

Certainly, thickness of electrode is another factor that can effectively change the power density due to the different resistance on the proton and O2 transport. We were trying to maintain the thickness of the coated electrodes as same as possible. The differences are the particles sizes and dispersibility of Pt/C and PpPD/XC72-Pt-MW (same amount of Nafion solution was added). They are found to be almost same from the TEM micrographs.

## 4. Conclusions

Based on avoiding the tedious and low yield Pt-preparing method of hydrothermal or solvothermal reduction, we prepared Pt-loading XC72 by MW irradiation in the presence of PpPD which is also an N-containing chemical that can effectively capture Pt ions and improve the yield of Pt.

The amino-groups of PpPD were found to convert to dihydrophenazine state under MW irradiation which inducing its redox reaction with Pt ions, as characterized by FTIR spectra. The increasing dihydrophenazine (secondary amine) and decreasing primary amines can be considered as the production of Pt elements confirmed by XPS. TEM micrographs show well-dispersed and tiny Pt particles on the XC72 and residue weight of TGA thermograms illustrated higher Pt-loading weight after MW irradiation in the presence of PpPD/XC72 composite. X-ray diffraction patterns indicate same type of Pt atoms similar to hydrothermal treatment method were loaded on XC72 and the particles size and active surface area are calculated as well. C-V diagram illustrates the significant Pt-H oxidation and Pt–O reduction behaviors assisted by MW irradiation in the presence of PpPD coated XC72 particles (PpPD/XC72). The LSV curves demonstrates the highest reduced current for PpPD/XC72-Pt-MW and its calculated number of e-transferred during ORR approaches 4 based on the calculation related to the K-L plots. The single cell performance demonstrates higher specific max. power density and maximum current density when Pt catalyst was prepared by facile MW irradiation in the presence of PpPD/XC72 nanocomposite.

The presence of primary amino-structure is found to be able to absorb MW and behave as both chelating and reducing agents during MW-assisted redox reaction. The simple way of MW irradiation can provide another choice to load Pt on the conducting support like XC72 to prepare Pt-catalyst electrode of MEA. In the future, we would ty other amino-containing compounds or polymers like melamine or chitosan as the MW absorber and active reducing agents for Pt reduction.

## Figures and Tables

**Table 1:** Various amino-groups of PpPD before and after MW irradiation from XPS.

Peak Area	–NH–	–NH_2_	–NH_3_^+^	–NH–/NH_2_
Neat PpPD (EB type)	520	1409	61	0.37
PpPD/XC72-Pt-MW	11384	11814	11592	0.96

**Table 2:** Particle size and electrochemical properties of Pt implanted in various supports.

Catalyst	d ^a^ (nm)	ESA ^b^ (cm^2^)	ECSA ^c^ (m^2^ g^−1^_Pt_)	MA ^d^ (mA mg^−1^_Pt_)
PpPD/XC72-Pt-MW	5.62	1103.0	60.1	739.2
Commercial Pt/C	4.38	680.1	30.5	484.5
XC72-Pt-MW	3.12	219.5	27.5	376.8
PpPD-Pt-MW	6.56	0.0	0	0

a: The average implanted Pt crystal size for each electrocatalyst electrode material is calculated by the Debye-Scherrer equation based on the X-ray diffraction (220) plane of Pt crystalline (Figure 5). $d=\frac{k\lambda }{\beta \cos\theta }$, where *k* is a coefficient (0.9), λ is the wavelength of the X-rays (0.1541nm for CuKα), β is the full-width half-maximum (FWHM) of the respective diffraction peak measured at 2θ (in radians) and θ is the diffraction angle of the peak in degree; b: ESA (cm^2^) = *Q*_H_ 0.21^−1^ (mC cm^−2^). Total hydrogen oxidation charge (Q_H_ (mC)) obtained from the integration area of H_2_ desorption (mAV) in C-V diagram (Figure 6) divided by scan speed (V s^−1^) 0.21: the theoretical, necessary charge to oxidize a monolayer of H_2_ on bright Pt; c: ECSA (m^2^ g^−1^) = ESA [Pt]^−1^, [Pt]: Weight of Pt in GC electrode = volume of slurry dropped on GC electrode × concentration (Pt-concentration in the slurry) × Pt % obtained from the residue weights of the corresponding Pt-catalysts demonstrated in TGA thermograms (Figure 4); d: MA (mass activity) = I (current density) × ECSA, I: obtained from LSV (1600 rpm) curves at 0.5 V (V_1/2_: half-wave potential) of Figure 7.

**Table 3:** Particle size and electrochemical properties of Pt implanted in various substrates.

Catalyst	^a^ Max. no. of e-Transferred	^b^ Loaded Pt wt %	^c^ Max. Power Density (mW cm^−2^)	^d^ Specific Power Density (mW cm^−2^_Pt_)
PpPD/XC72-Pt-MW	3.83	18.49	639.7	34.6
Commercial Pt/C	3.62	22.30	684.8	30.6
XC72-Pt-MW	3.75	16.66	350.2	21.0
PpPD-Pt-MW	-	4.05	5.0	1.2

a: obtained from Figure 7 based on the calculation from Koutecky-Levich (K-L) analysis; b: obtained from the residue wt % of Figure 4; c: obtained from the max. value of power density in Figure 8; d: obtained from the ratio of max. power density/loaded Pt wt %.
